# Safety, Tolerability, and Pharmacokinetics of a Novel Nitroimidazooxazole Antitubercular Agent in Healthy Adults: A Phase I Trial

**DOI:** 10.1002/mco2.70510

**Published:** 2025-12-04

**Authors:** Jia Miao, Zhenling Wang, Zhenyu Ding, Huashan Shi, Yongping Qin, Tiantao Gao, Ning Jiang, Jianqing He, Manni Wang, Xiawei Wei

**Affiliations:** ^1^ GCP Center/Institute of Drug Clinical Trials West China Hospital Sichuan University Chengdu Sichuan China; ^2^ Laboratory of Aging Research and Cancer Drug Target National Clinical Research Center for Geriatrics State Key Laboratory of Biotherapy and Cancer Center West China Hospital Sichuan University Chengdu China; ^3^ Department of Biotherapy Cancer Center and State Key Laboratory of Biotherapy West China Hospital Sichuan University Chengdu Sichuan China; ^4^ Jumbo Drug Bank Co., Ltd Chengdu China; ^5^ Department of Pulmonary and Critical Care Medicine State Key Laboratory of Respiratory Health and Multimorbidity West China Hospital Sichuan University Chengdu China

**Keywords:** anti‐tuberculosis, clinical trial, JBD0131, safety

## Abstract

This study evaluated the safety, tolerability, and pharmacokinetics of JBD0131, a novel nitroimidazooxazole antitubercular agent, in healthy adults. We previously reported JBD0131, a novel nitroimidazooxazole antitubercular agent, which overcomes drug resistance and bioavailability limitations of existing anti‐tuberculosis therapies. The clinical trial was structured into three parts: an initial single ascending dose (SAD) phase under fasting conditions, a food‐effect assessment, and a final multiple ascending dose (MAD) phase conducted after meals.  Among 95 enrolled participants, JBD0131 demonstrated favorable safety and tolerability across all regimens. No serious adverse events (AEs) or treatment discontinuations occurred. Treatment‐emergent AE incidence was comparable to placebo without dose‐dependent trends. Pharmacokinetic (PK) analysis showed that systemic exposure for JBD0131, measured by maximum plasma concentration (*C*_max_) and area under the plasma concentration–time curve (AUC), increased proportionally with the dose. The presence of food significantly enhanced the bioavailability and delayed the median time to reach peak concentration (*T*_max_) by approximately 2 h. These findings collectively demonstrate that JBD0131 has an acceptable safety profile and predictable, linear pharmacokinetics in healthy adults. The observed food effect, which boosts systemic exposure, along with the drug's linear accumulation, supports the need for further investigation to define optimal treatment regimens for future clinical development.

## Graphical abstract

Mean blood drug concentration–time curves (linear and semi‐logarithmic) of JBD0131 in the (A) single‐dose group; (B) food‐affected group.

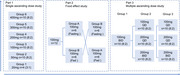

## 1 Introduction

According to the Global Tuberculosis Report 2024 [1], it causes approximately 10.8 million new cases in 2023. Most TB cases in 2023 occurred in South‐East Asia (45%), and Africa (24%), followed by Western Pacific (17%). An estimated 1.09 million deaths were reported globally among HIV‐negative people in 2023. Though this data is lower than that of 2019, the reduction in the number of TB‐caused deaths between 2015 and 2023 was 23%, which fails to reach the 2025 milestone of the WHO End TB Strategy [2]. Furthermore, advances in global TB programs over the last decade have been severely undermined by COVID‐19, most notably in a global 18% decline in new TB cases diagnosed between 2019 and 2020.

The causative pathogen, *Mycobacterium tuberculosis*, is transmitted via aerosolized droplets and primarily infects the lungs, where it evades host immunity by residing within alveolar macrophages [3, 4]. FDA‐approved medications for *Mycobacterium tuberculosis* infections include rifampin, isoniazid, pyrazinamide, and ethambutol [5]. Drug‐resistant tuberculosis accounts for a notable proportion of the global antimicrobial resistance (AMR) burden [6, 7]. Monoresistance to rifampicin or isoniazid is a growing concern in TB endemic regions, with 71% of pulmonary TB cases confirmed with rifampicin resistance in 2020 [8]. A more severe complication of tuberculosis medications is multi‐drug‐resistant tuberculosis (MDR‐TB), which is distinguished from monoresistance to first‐line therapies isoniazid and rifampin [9]. Second‐line drugs should be used when drug resistance develops to the first‐line agents, which include kanamycin, capreomycin, amikacin, and fluoroquinolones such as levofloxacin, moxifloxacin, and gatifloxacin [10, 11]. A unique and more lethal type of MDR‐TB is extensively multi‐drug‐resistant tuberculosis (XDR‐TB), characterized by resistance to first‐line rifampin and isoniazid, one second‐line aminoglycoside, and either of the fluoroquinolones [12, 13]. Moreover, the suggested first‐line regimens require 6–9 months of multi‐drug treatment, which contributes to high rates of non‐adherence and drug‐related toxicities that frequently lead to therapy interruption.

Drugs that have recently received FDA approval for multi‐drug resistance TB are pretomanid, used in sequence with bedaquiline and linezolid [14, 15, 16]. While delamanid, a nitroimidazole analog, remains unapproved by the FDA for MDR‐TB, it shows greater potency than pretomanid in reducing mycobacterial burden at reduced concentrations [17, 18]. However, its regulatory status and pharmacokinetic profile present challenges for widespread use. Both compounds demonstrate poor oral bioavailability owing to low aqueous solubility [19]. Moreover, the adverse effect of pretomanid such as peripheral neuropathy and anemia also limited its further application, along with complex drug–drug interactions that complicate their integration into broader MDR‐TB regimens [18].

These limitations lead to the need for novel anti‐tuberculosis agents that combine robust efficacy with favorable safety and pharmacokinetic properties. Our prior work reported novel nitroimidazooxazoles, structurally related to delamanid but more potent than pretomanid [20]. We aimed to retain the antimycobacterial efficacy of delamanid while significantly enhancing drug‐like properties for improved oral absorption and clinical outcomes. The optimization of a compound 1 from a series of heterobicyclic nitroimidazooxazoles with potent in vitro activity yielded clinical candidate JBD0131. As a nitroimidazole, JBD0131 was specifically designed to overcome the key limitations of its predecessors, namely to achieve enhanced oral bioavailability and a reduced adverse effect profile. To accelerate its clinical translation, this Phase I study systematically evaluated JBD0131 safety and pharmacokinetics in healthy Chinese adults. In this trial, a single ascending dose trial defined its tolerability range, a two‐period crossover design assessed food effects on pharmacokinetics, and a multiple‐dose study simulated clinical exposure. These pivotal data will inform efficacy trials in tuberculosis participants, facilitating optimized regimen development with significant public health implications.

## 2 Results

### 2.1 Patient Characteristics

**FIGURE 1:**
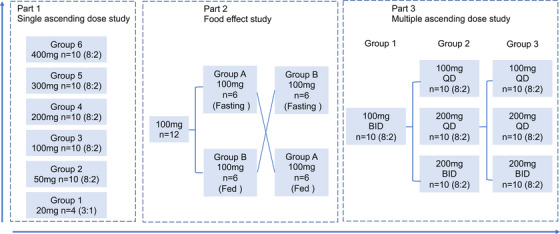
Flowchart of the Phase I study.

A total of 182 healthy volunteers were screened across this clinical pharmacology program: 10 for the pilot bioanalytical cohort and 172 for the main study. Ultimately, 95 participants were enrolled: 8 in the pilot cohort and 87 in the main study comprising single ascending dose (SAD), food effect (FE), and multiple ascending dose (MAD) phases. Primary reasons for screening failure (*n* = 87) included protocol‐defined exclusion criteria (*n* = 71) and withdrawn consent (*n* = 13). Demographic characteristics (age, sex, height, weight, BMI, ethnicity, fertility status) were balanced across all SAD, FE, and MAD dose cohorts. The study flowchart is shown in Figure 1.

In the single‐dose, dose‐escalation study, a total of 54 participants were enrolled. Among them, 51 participants (94.4%) were of Han ethnicity, while the remaining 3 participants (5.6%) were of other ethnicities. The cohort comprised 30 males (55.6%) and 24 females (44.4%). The age range of the participants was from 18 to 39 years. The median age of the participants were 23.5 years and the median baseline body mass index (BMI) were 21.75 kg/m^2^. Among the 12 participants enrolled in the food effect study, 8 were male (66.7%) and 4 were female (33.3%). The age range was from 20 to 31 years. The 21 participants enrolled in the multiple ascending dose study included 12 males (57.1%) and 9 females (42.9%), ranging from 19 to 34 years. The demographic and baseline characteristics of the participants in the SAD, food effect, and MAD study are summarized in Table 1.

**TABLE 1:** The demographic and baseline characteristics of the participants in the single ascending dose study, food‐effect study, and multiple ascending dose study.

Single ascending dose (SAD) study									Food effect study			Multiple ascending dose (MAD) study			
	**20 mg**	**50 mg**	**100 mg**	**200 mg**	**300 mg**	**400 mg**	**Placebo**	**Total**	**Fasting to Postprandial**	**Postprandial to Fasting**	**Total**	**100 mg BID**	**200 mg BID**	**Placebo**	**Total**
	**(*N* = 3)**	**(*N* = 8)**	**(*N* = 8)**	**(*N* = 8)**	**(*N* = 8)**	**(*N* = 8)**	**(*N* = 11)**	**(*N* = 54)**	**(*N* = 6)**	**(*N* = 6)**	**(*N* = 12)**	**(*N* = 8)**	**(*N* = 8)**	**(*N* = 5)**	**(*N* = 21)**
**Age (years)**															
Mean	28.7	26.1	23.8	27	23.9	24.8	24	25.1	24.7	24.3	24.5	24	26	24.2	24.8
SD	6.43	4.94	5.09	6.72	3.48	4.53	4.71	4.99	2.16	4.63	3.45	5.29	4.81	2.49	4.48
Median	26	23.5	22	25	23	24	22	23.5	24.5	23	23.5	22.5	26	25	24
Min, Max	24, 36	22, 34	18, 35	19, 39	21, 31	18, 34	19, 34	18, 39	22, 28	20, 31	20, 31	19, 34	20, 34	20, 26	19, 34
**Gender *n* (%)**															
Male	1 (33.3)	4 (50.0)	5 (62.5)	4 (50.0)	4 (50.0)	5 (62.5)	7 (63.6)	30 (55.6)	4 (66.7)	4 (66.7)	8 (66.7)	5 (62.5)	5 (62.5)	2 (40.0)	12 (57.1)
Female	2 (66.7)	4 (50.0)	3 (37.5)	4 (50.0)	4 (50.0)	3 (37.5)	4 (36.4)	24 (44.4)	2 (33.3)	2 (33.3)	4 (33.3)	3 (37.5)	3 (37.5)	3 (60.0)	9 (42.9)
**Ethnicity *n* (%)**															
Han	3 (100)	8 (100)	7 (87.5)	8 (100)	7 (87.5)	8 (100)	10 (90.9)	51 (94.4)	6 (100)	6 (100)	12 (100)	8 (100)	8 (100)	5 (100)	21 (100)
Others	0	0	1 (12.5)	0	1 (12.5)	0	1 (9.1)	3 (5.6)	0	0	0	0	0	0	0
**Height (cm)**															
Mean	159.67	166.51	166.85	163.78	162.54	165.54	169.29	165.61	167.52	171.6	169.56	162.2	167.98	158.46	163.51
SD	8.949	9.973	6.963	8.537	9.035	7.911	7.51	8.305	5.348	7.951	6.803	7.579	11.937	7.233	9.771
Median	154.5	163.55	166.2	161.65	165.35	165.55	170.2	165.75	167.8	172	168.8	164.15	170.9	157.5	163.6
Min, Max	154.5, 170.0	155.0, 185.5	153.7, 177.7	153.3, 175.6	147.7, 171.7	152.0, 176.1	157.9, 179.3	147.7, 185.5	159.0, 174.5	163.0, 182.3	159.0, 182.3	150.4, 170.0	150.1, 184.9	150.8, 169.1	150.1, 184.9
**Weight (kg)**															
Mean	55.77	60.93	60.09	60.09	56.55	57.33	61.88	59.4	59.02	57.93	58.48	58.1	61.04	52.88	57.98
SD	7.051	10.233	7.634	10.267	8.137	8.27	7.156	8.316	8.764	5.749	7.089	5.829	9.085	5.879	7.612
Median	55.6	57.8	63.8	62.55	56.75	56.45	63.4	60.05	60.5	57.15	58.5	60.75	61.2	50.8	59.3
Min, Max	48.8, 62.9	49.3, 76.4	47.4, 68.2	45.0, 72.8	45.1, 69.6	45.1, 68.0	48.2, 72.0	45.0, 76.4	46.9, 69.0	50.0, 64.6	46.9, 69.0	49.8, 64.0	49.3, 74.9	49.4, 63.3	49.3, 74.9

### 2.2 Safety

#### 2.2.1 Single Ascending Dose (SAD) Safety Study

All 54 participants receiving JBD0131 or placebo in the SAD study were evaluated for safety. Thirty participants (55.6%) reported a total of 54 treatment‐emergent adverse events (TEAEs). No serious adverse events (SAEs) occurred, and no participants discontinued treatment due to adverse events (AEs). TEAE incidence across cohorts showed no dose‐dependent trend: 0% in the 20 mg group, 62.5% (12 events) in the 50 mg group, 75.0% (12 events) in the 100 mg group, 62.5% (8 events) in the 200 mg group, 62.5% (10 events) in the 300 mg group, and 37.5% (3 events) in the 400 mg group. Moreover, placebo recipients exhibited comparable incidence of TEAEs (54.5%; 9 events). Table S1 summarized the treatment‐related adverse events observed in the single‐dose escalation study.

All participants reported laboratory abnormalities to some degree, with clinically significant abnormalities documented in 25 participants (46.3%). The most prominent clinically relevant change of treatment versus placebo included elevated urinary erythrocytes, urinary occult blood positivity, increased serum creatine kinase (CK) and amylase, and decreased hemoglobin (Hb). Neither ophthalmological examinations, Hamilton Depression Scale‐scored ≥7, vital signs or ECGs showed clinically concerning trends.

#### 2.2.2 Food Effect Safety Study

All 12 participants administered JBD0131 under fasting and fed conditions reported 25 TEAEs in total. No serious adverse events (SAEs) occurred, and no participants discontinued treatment due to AEs. TEAE incidence was comparable between conditions: 8 participants (66.7%) reported 11 events during fasting periods versus 9 participants (75.0%) reporting 14 events during fed periods. TRAEs occurred in 11 participants (91.7%), comprising 20 events, the most frequent of which were occult blood positive, lipase increased, cortisol decreased, and headache (Table S2). Laboratory assessments revealed expected pharmacokinetic interactions with no clinically concerning trends. Of the 12 subjects who received the study drug, all 12 (100%) reported a total of 752 abnormal laboratory findings after administration. Among these, 11 subjects (91.7%) reported 62 abnormal laboratory findings of clinical significance. The incidence of clinically significant abnormal laboratory findings was generally consistent between fasting and fed conditions (Table S3).

#### 2.2.3 Multiple Ascending Dose (MAD) Safety Study

TEAE incidence was 100% across all cohorts: 25 events in the 100 mg BID group (*n* = 8), 32 events in the 200 mg BID group (*n* = 8), and 8 events in the placebo group (*n* = 4). Treatment‐related adverse events (TRAEs) occurred in 17 participants (85.0%), comprising 44 events (Table S4). No dose‐dependent increase in TEAE or TRAE frequency was observed. The most prominent clinically significant laboratory abnormalities versus placebo included increased serum creatinine (Cr), lipase, alanine aminotransferase (ALT), fecal occult blood positivity, cortisol, amylase, aspartate aminotransferase (AST), gamma‐glutamyltransferase (GGT), CK, erythrocytosis, and decreased hemoglobin. Taken together, JBD0131 demonstrated favorable safety and tolerability profiles in healthy Chinese adults across all evaluated regimens: single doses (20–400 mg) under fasting conditions, food‐effect assessment, and multiple doses (100–200 mg BID) after fed conditions.

### 2.3 Pharmacokinetics

#### 2.3.1 Pharmacokinetics of JBD0131

Following single‐dose administration of JBD0131, median time to maximum plasma concentration (*T*_max_) of JBD0131 ranged between 2.00 and 3.00 h. All dose groups demonstrated similar plasma concentration–time profiles. Representative mean plasma concentration–time curves, and mean pharmacokinetic parameters are presented in Table 2, respectively. The maximum plasma concentration (*C*_max_) and AUC (area under the plasma concentration–time curve) values increased proportionally with dose escalation from 50 to 400 mg except at the 300 mg dose level. Clearance (*CL*/*F*) and apparent volume of distribution (*V_d_*/*F*) values were comparable between 50 and 200 mg cohorts, while significantly higher parameters were observed at 300 mg and 400 mg doses.

**TABLE 2:** Summary of pharmacokinetic parameters of WXWH0131 in participants in the single‐dose group and food‐effect dose group.

		Single ascending dose (SAD) study					Food effect study	
PK			50 mg	100 mg	200 mg	300 mg	Fasting state	Postprandial state
parameters			(*N* = 8)	(*N* = 8)	(*N* = 8)	(*N* = 8)	(*N* = 12)	(*N* = 12)
*C*_max_ (ng/mL)		Mean ± SD	108.3713 ± 44.9665	229.9168 ± 95.3647	320.2854 ± 138.1711	260.2776 ± 96.8072	210.5976 ± 81.3369	366.2343 ± 117.3022
*T*_max_ (h)		Median (Min, Max)	2.00 (1.50, 4.00)	2.25 (1.50, 4.00)	3.00 (2.00, 4.00)	2.00 (1.50, 5.03)	2.50 (1.50, 3.50)	4.50 (3.50, 6.00)
AUC_0–12_ (h*ng/mL)		Mean ± SD	750.5203 ± 362.1303	1535.4615 ± 529.1420	2373.6594 ± 1096.4228	1810.5394 ± 628.3486	1450.9566 ± 614.3759	2660.7042 ± 730.9040
AUC_0–96_ (h*ng/mL)		Mean ± SD	1290.9920 ± 639.5585	2594.9213 ± 7 47.3679	5098.7059 ± 2285.7738	4248.6778 ± 1799.7828	2667.5211 ± 1257.9936	5761.5730 ± 1770.0496
AUC_0–_ *_t_* (h*ng/mL)		Mean ± SD	1264.9723 ± 640.5738	2569.6424 ± 733.2642	5095.9319 ± 2299.6648	4255.4837 ± 1821.5362	2646.0932 ± 1264.8283	5736.3166 ± 1764.0724
AUC_0–∞_ (h*ng/mL)		Mean ± SD	1297.0270 ± 642.4847	2610.1886 ± 757.0263	5136.8330 ± 2298.7910	4281.8218 ± 1821.2734	2676.4930 ± 1263.4572	5780.5857 ± 1775.2862
*t*_1/2_ (h)		Mean ± SD	10.15 ± 4.03	9.83 ± 4.54	12.29 ± 3.21	11.74 ± 3.16	10.00 ± 2.90	10.40 ± 2.64
*V_d_*/*F* (L)		Mean ± SD	746.3819 ± 517.6706	556.7667 ± 224.3551	834.7498 ± 407.6632	1324.2951 ± 583.0676	668.9351 ± 428.2025	279.9179 ± 104.0506
*CL*/*F* (L/h)		Mean ± SD	54.4758 ± 43.3470	40.9604 ± 43.3470	51.0357 ± 43.3470	81.4443 ± 43.3470	48.8837 ± 31.7009	18.7626 ± 5.4536
*λ_z_* (1/h)		Mean ± SD	0.0766 ± 0.0246	0.0853 ± 0.0396	0.0599 ± 0.0158	0.0656 ± 0.0296	0.0751 ± 0.0229	0.0702 ± 0.0158
MRT (h)		Mean ± SD	12.96 ± 4.02	13.75 ± 5.64	18.28 ± 3.22	20.01 ± 4.62	14.25 ± 3.63	17.43 ± 2.89

Abbreviations: pharmacokinetics (PK), maximum concentration (*C*_max_), time to maximum concentration (*T*_max_), area under the concentration–time curve (AUC), area under the curve from 0 to 12 h (AUC_0–12_), area under the curve from 0 to 96 h (AUC_0–96_), area under the curve from 0 to the last measurable concentration (AUC_0–_ *_t_*), area under the curve from 0 to infinity (AUC_0–∞_), half‐life (*t*_1/2_), volume of distribution divided by bioavailability (*V_d_*/*F*), clearance divided by bioavailability (*CL*/*F*), elimination rate constant (*Ke*), mean residence time (MRT).

**FIGURE 2:**
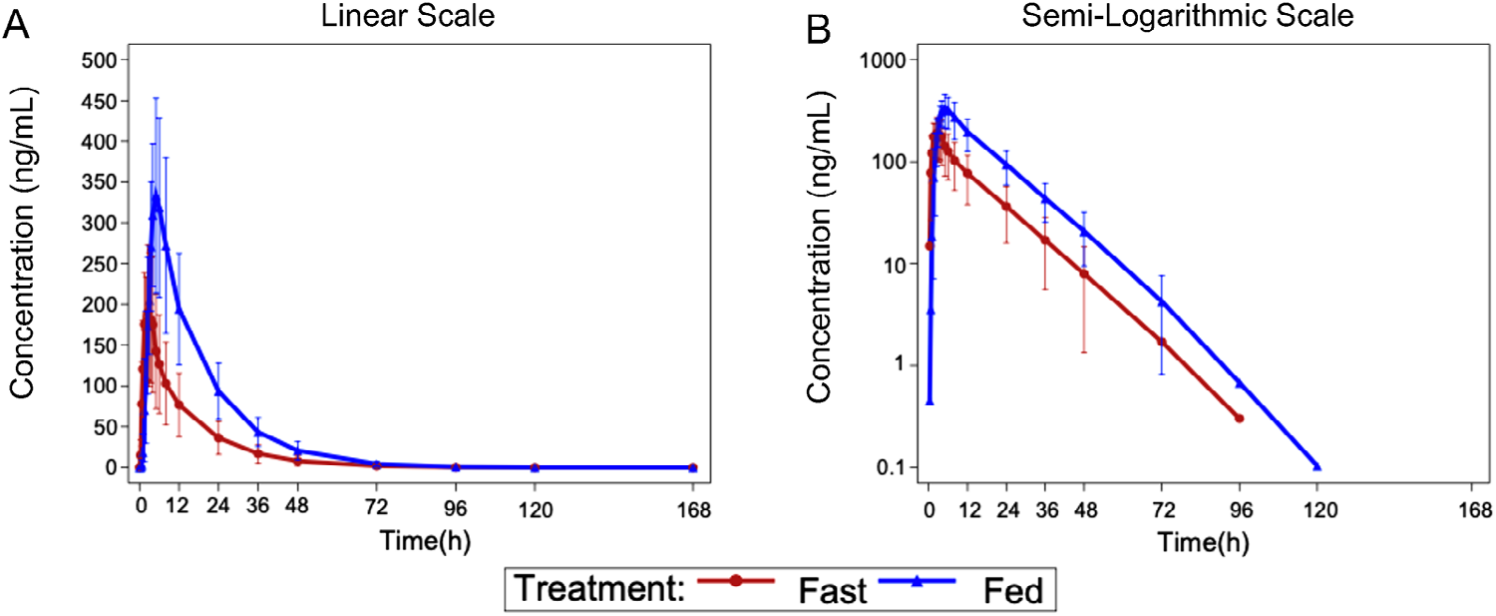
Mean blood drug concentration–time curves (A, linear and B, semi‐logarithmic) of JBD0131 in the food‐effect group.

Following single‐dose administration of 100 mg JBD0131, the mean plasma concentration of JBD0131 was significantly higher under fed conditions compared to fasting conditions (Figure 2A, B). The median *T*_max_ values for JBD0131 was 2.50 h under fasting and 4.50 h under fed conditions, respectively, indicating a 2‐h delay in *T*_max_ under fed conditions compared to fasting. Additionally, the *CL*/*F* and *V_d_*/*F* values for JBD0131 were lower under fed conditions compared to fasting, whereas the average *t*_1/2_ and *λz* values were similar between the two conditions (Table 2).

**FIGURE 3:**
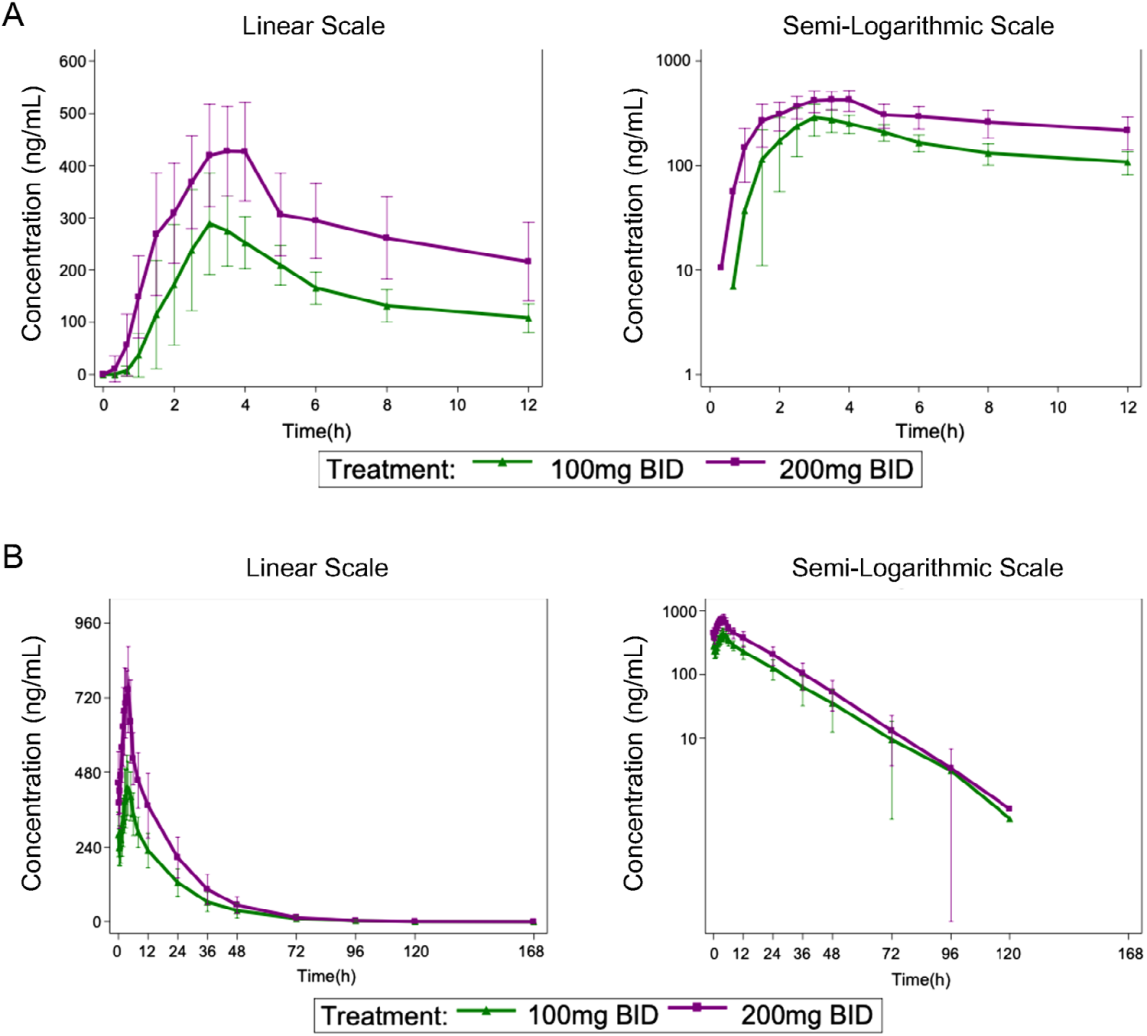
Mean blood drug concentration–time curves (linear and semi‐logarithmic) of JBD0131 in the multiple‐dose group on (A) Day 1 and (B) Day 14.

In the multiple‐dose study, patients received JBD0131 orally at two different dosing regimens (100 mg BID and 200 mg BID). Both Day 1 (Figure 3A) and Day 14 (Figure 3B) exhibited comparable concentration–time profiles at each dose level, with mean plasma concentrations reaching peak levels at approximately 3 h post‐dose. On Day 1, the median *T*_max_ values for JBD0131 were 3.25 h in both dose groups. After the first dose of JBD0131 in the multiple‐dosing trial, the mean *C*_max_ values were 302.8505 ng/mL and 458.9593 ng/mL, and the mean AUC_0–12_ values were 1811.3126 h·ng/mL and 3251.4226 h·ng/mL for the 100 mg BID and 200 mg BID groups, respectively. These results indicate that *C*_max_ and AUC of JBD0131 on Day 1 increased with the dose escalation (Table S5). After 14 days of multiple dosing with JBD0131 at 100 mg BID and 200 mg BID, the terminal half‐life (*t*_1/2_), CL*/F*, *ss* (*CL*/*F* steady state), and *V_d_*/*F*, *ss* (*V_d_*/*F* steady state) values were similar between the two groups (Table S6). These results demonstrate that after 14 days of continuous dosing with JBD0131 at 100 mg BID and 200 mg BID, there was approximately a 1.5‐ to 2.1‐fold accumulation of JBD0131 in plasma.

#### 2.3.2 Pharmacokinetics of Metabolite WXWD

The comprehensive pharmacokinetic characterization of the metabolite WXWD not only fulfills regulatory requirements but also provides crucial insights into the overall pharmacological profile of JBD0131. In the single‐dose study, participants received a single oral dose of JBD0131 at five different dose levels. The median *T*_max_ values for WXWD were 2.75, 3.00, 3.50, 3.00, and 3.50 h for the 50, 100, 200, 300, and 400 mg dose groups, respectively, indicating that the median *T*_max_ values were relatively similar across the dose groups (Table 3). All cohorts exhibited similar concentration–time profiles, with peak concentrations occurring at approximately 3 h post‐dose.

**TABLE 3:** Summary of pharmacokinetic parameters of WXWD in participants in the single‐dose group and food‐effect dose group.

		Single ascending dose (SAD) study					Food effect study	
PK		50 mg	100 mg	200 mg	300 mg	400 mg	Fasting state	Postprandial state
parameters		(*N* = 8)	(*N* = 8)	(*N* = 8)	(*N* = 8)	(*N* = 8)	(*N* = 12)	(*N* = 12)
*C*_max_ (ng/mL)	Mean ± SD	11.8336 ± 5.5046	36.7934 ± 14.5410	36.1188 ± 16.0915	27.8411 ± 9.4079	40.9808 ± 19.5466	20.0786 ± 9.3785	35.5263 ± 12.8198
*T*_max_ (h)	Median (Min, Max)	2.75 (1.50, 5.00)	3.00 (2.00, 3.00)	3.50 (2.00, 4.00)	3.00 (2.00, 12.02)	3.50 (2.00, 5.00)	3.75 (2.50, 4.00)	5.00 (4.00, 8.00)
AUC_0–12_ (h·ng/mL)	Mean ± SD	84.7142 ± 44.6603	226.8492 ± 84.9220	239.2738 ± 106.1344	208.9331 ± 69.3799	263.4087 ± 120.1002	150.0149 ± 65.7672	263.1118 ± 81.1032
AUC_0–96_ (h·ng/mL)	Mean ± SD	230.1827 ± 124.2819	583.2692 ± 187.2728	858.6262 ± 375.0142	871.7829 ± 368.3770	1103.5318 ± 537.8984	511.4681 ± 250.5536	1110.8005 ± 403.4350
AUC_0–_ *_t_* (h·ng/mL)	Mean ± SD	229.6475 ± 125.1696	583.2692 ± 187.2728	1017.2792 ± 455.8404	1031.1396 ± 462.2884	1334.7231 ± 679.9166	612.1549 ± 313.1013	1316.7217 ± 495.4314
AUC_0–∞_ (h·ng/mL)	Mean ± SD	261.9847 ± 136.9037	667.5329 ± 200.3672	1162.3085 ± 542.7045	1148.5013 ± 558.7732	1498.7554 ± 806.6787	719.5893 ± 390.6755	1480.1406 ± 565.4758
*t*_1/2_ (h)	Mean ± SD	33.83 ± 10.88	36.57 ± 12.80	59.78 ± 16.30	46.96 ± 16.55	52.13 ± 8.50	66.93 ± 21.90	59.28 ± 16.08
*λ_z_* (1/h)	Mean ± SD	0.0226 ± 0.0079	0.0208 ± 0.0062	0.0127 ± 0.0049	0.0167 ± 0.0064	0.0136 ± 0.0024	0.0114 ± 0.0037	0.0126 ± 0.0036
MRT (h)	Mean ± SD	26.50 ± 4.56	26.25 ± 5.25	45.96 ± 6.25	46.79 ± 11.93	50.93 ± 4.82	46.05 ± 8.82	48.03 ± 5.73

Abbreviations: pharmacokinetics (PK), maximum concentration (*C*_max_), time to maximum concentration (*T*_max_), area under the concentration–time curve (AUC), area under the curve from 0 to 12 h (AUC_0–12_), area under the curve from 0 to 96 h (AUC_0–96_), area under the curve from 0 to the last measurable concentration (AUC_0–_ *_t_*), area under the curve from 0 to infinity (AUC_0–∞_), half‐life (*t*_1/2_), volume of distribution divided by bioavailability (*V_d_*/*F*), clearance divided by bioavailability (*CL*/*F*), elimination rate constant (*Ke*), mean residence time (MRT).

**FIGURE 4:**
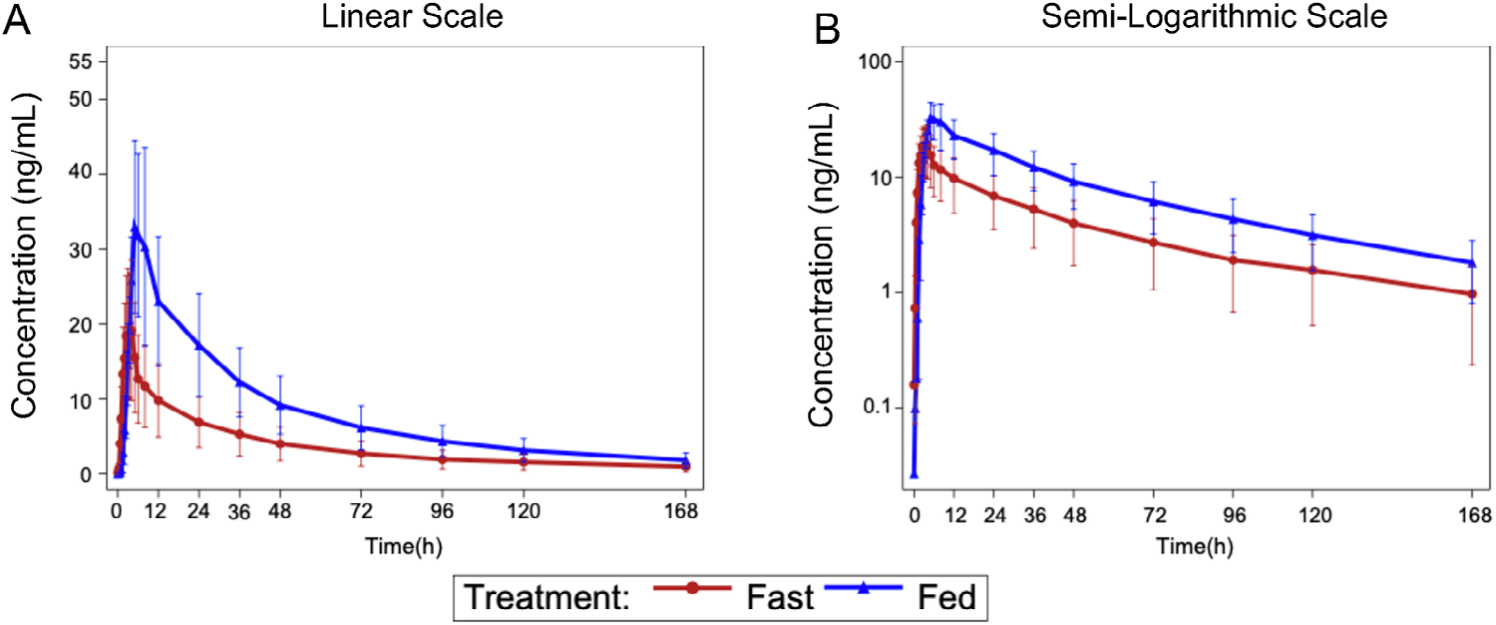
Mean blood drug concentration–time curves (A, linear and B, semi‐logarithmic) of WXWD in the food‐ effect group.

Following single oral administration of 100 mg JBD0131, the plasma exposure levels of the metabolite WXWD, measured by *C*_max_, AUC_0–12_, AUC_0–96_, AUC_0–_ *_t_*, and AUC_0–∞_, were higher under fed conditions than under fasting conditions (Figure 4A, B). The median *T*_max_ values for the metabolite WXWD were 3.75 h under fasting conditions and 5.00 h under fed conditions, indicating a 1.25‐h delay in *T*_max_ under fed conditions compared to fasting. The mean *λz* values for the metabolite WXWD were similar between fasting and fed conditions, whereas the mean *t*_1/2_ value was approximately 8 h shorter under fed conditions (Table 3).

**FIGURE 5:**
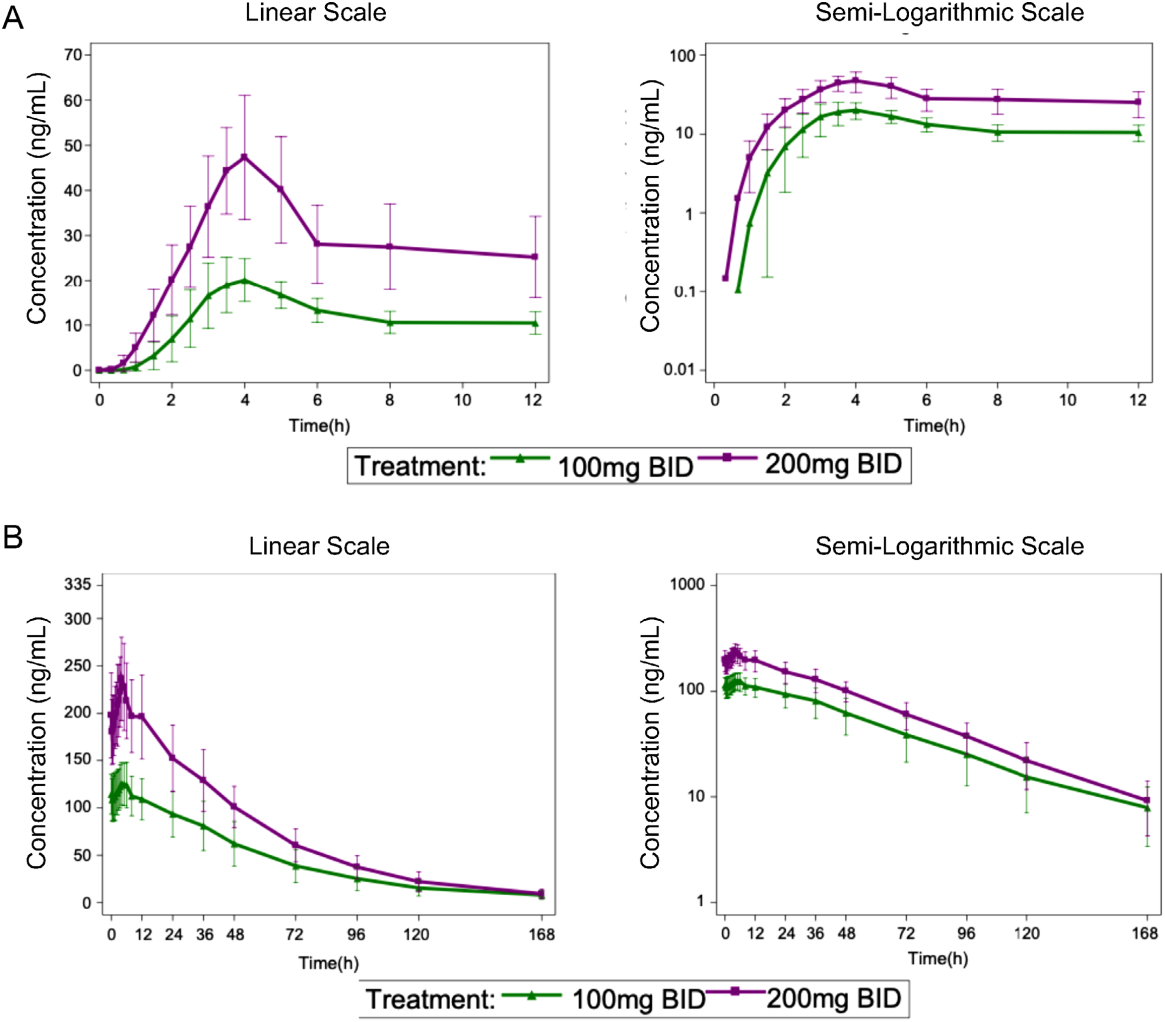
Mean blood drug concentration–time curves (linear and semi‐logarithmic) of WXWD in the multiple‐dose group on (A) Day 1 and (B) Day 14.

In the multiple‐dose study, Day 1 (Figure 5A) and Day 14 (Figure 5B) exhibited similar concentration–time profiles, consistently achieving peak levels at 4 h post‐dose. After the first oral administration of JBD0131, the median *T*_max_ values for WXWD on Day 1 were 4.00 h and 3.75 h for the 100 mg BID and 200 mg BID dose groups, respectively. Following the initial dose of JBD0131 in the multiple‐dosing trial, *C*_max_ and AUC of WXWD increased with the dose escalation (Table S7). On Day 14, plasma exposure levels of WXWD (*C*_max_, AUC0–12, *ss*, AUC0–*t*, *ss*, and AUC_0–∞_, *ss*) also increased with the dose (Table S8). Steady‐state conditions were evidenced by minimal concentration fluctuations between D11 and D14 for both 100 mg and 200 mg BID regimens, confirming D14 pharmacokinetic parameters can be used to evaluate the steady‐state characteristics of WXWD.

## 3 Discussion

Tuberculosis remains a global health problem, with multidrug‐resistant strains posing urgent therapeutic challenges. *Mycobacterium tuberculosis* is characterized by its ability to manipulate host immune responses, forming granulomas that both contain and potentially facilitate bacterial persistence. The pathogenesis of human tuberculosis involves complex interactions between hosts and virulence factors for survival and immune escapes of *Mycobacterium tuberculosis* [4, 21, 22]. Treatment for drug‐resistant TB has evolved in recent decades, notably switching to oral regimens and the short‐term (6 months) regimens for rifampin‐resistant TB and MDR‐TB [23].

JBD0131, a next‐generation nitroimidazole specifically designed to overcome the limitations of delamanid and pretomanid, demonstrated an acceptable safety profile across single‐dose (20–400 mg) and multi‐dose (100–200 mg BID for 14 days) regimens. This first‐in‐human study evaluated its safety, pharmacokinetics, and food effects in healthy adults. Based on this favorable safety and tolerability profile, we recommend that Phase II clinical trials explore dose‐response relationships using doses up to and including 200 mg BID. Although adverse events (AEs) were common, most were mild, with no SAEs or withdrawals in any cohort. Drug‐related AEs (ADRs) were typically transient, and incidence did not escalate with dose. Importantly, AE rates mirrored placebo in controlled cohorts, supporting preliminary tolerability. Furthermore, the significant systemic exposure to WXWD observed across all dosing regimens, coupled with its prolonged presence, strongly suggests that WXWD is not an inactive byproduct but a pharmacologically relevant entity. Regarding its potential contribution to efficacy, the sustained plasma concentrations of WXWD, which demonstrated accumulation upon multiple dosing, may underpin a continuous pharmacological effect.

The pronounced food effect was observed that high‐fat meals increased systemic exposure of JBD0131 and its active metabolite WXWD versus fasting. From a safety perspective, the altered pharmacokinetics of WXWD under fed conditions warrants the monitoring of WXWD levels and postprandial dosing in future trials. The food‐effect assessment demonstrates that administration of JBD0131 with food significantly alters the pharmacokinetic profile of both the parent drug and its metabolite WXWD, without substantially impacting the safety and tolerability. The similar incidence of TEAEs between fasting and fed conditions indicate that the safety profile remains acceptable under both conditions. Pharmacokinetically, the higher plasma concentrations of JBD0131 under fed conditions, as well as reduced *CL*/*F* and *V_d_*/*F* values and delayed *T*_max_, suggest that food enhances the oral bioavailability of JBD0131, potentially through improved dissolution or reduced first‐pass metabolism. A similar pattern was observed for the metabolite WXWD, which showed decreased *t*_1/2_ of WXWD under fed state. These results collectively support that JBD0131 can be administered without strict fasting requirements, providing flexibility for clinical dosing.

The nitroimidazole anti‐tuberculosis drugs JBD0131, pretomanid, and delamanid all belong to the same chemical class, but possess pharmacokinetic characteristics. Following single oral administration, the median *T*_max_ of JBD0131 ranged from 2.00 to 3.00 h, whereas food intake delayed the *T*_max_ to 4.5 h but significantly increased systemic exposure, suggesting enhanced oral bioavailability under fed conditions. In contrast, *T*_max_ for delamanid is approximately 4 h. Food intake can increase its bioavailability by 2‐ to 4‐fold [24]. Pretomanid reaches peak plasma concentrations around 2‐4 h post‐dose. Food also significantly enhances its systemic exposure, but effective exposure levels are maintained under fed conditions [25]. Following multiple‐dose administration, JBD0131 demonstrates a relatively short half‐life with an accumulation factor of approximately 1.5‐ to 2.1‐fold over 14 days, suggesting rapid clearance and low accumulation potential. For delamanid, which shows a predicted terminal half‐life of 15.1 h, its metabolite DM‐6705 exhibits an extended half‐life of 7.8 days, respectively, indicating potential accumulation during prolonged therapy and raising concerns about QT prolongation after weeks to months of treatment [26]. Thus, the pharmacokinetic profile of JBD0131 characterized by rapid clearance, low accumulation potential, and a significant food‐enhanced exposure, collectively stand in contrast to delamanid which demonstrates a prolonged half‐life and high accumulation risk, and also to pretomanid, the exposure of which is affected by CYP3A4 inducers [27]. These distinctions provide an important basis for clinical dose optimization, and future combination therapy strategies of JBD0131.

While this Phase I study demonstrated a favorable safety and tolerability profile for JBD0131 in healthy subjects over 14 days, it is crucial to acknowledge that the relatively short duration of exposure precludes definitive conclusions on long‐term safety. Adverse events and organ toxicity associated with cumulative exposure may only be apparent until longer‐term administration. A comprehensive safety assessment of JBD0131 will require rigorous evaluation in Phases II and III trials involving TB patients over the full course of treatment. Furthermore, the comparable *C*_max_ and AUC of JBD0131 and its metabolite WXWD observed between the 300 and 200 mg dose groups may be attributed to two mechanisms. First, the observed decrease in exposure at 300 mg followed by an increase at 400 mg suggests a potential saturation of a clearance pathway within this dose range. At lower doses (50–200 mg), the metabolic enzymes responsible for the clearance of JBD0131 are not saturated. As the dose increases to 300 mg, the capacity of these enzymes is approached or saturated. Secondly, 300 mg dose of JBD0131 potentially exceeds the maximum transport capacity of active transporters, resulting in reduced absorption efficiency. Last but not least, the 300 mg dose may surpass the maximum solubility of JBD0131, preventing dose‐linear absorption. This interpretation is supported by our food‐effect study results, where a high‐fat diet significantly increased systemic exposure, suggesting that drug absorption relies on bile acid‐mediated or lipid‐based absorption, a property that could be leveraged to enhance bioavailability in malnourished TB patients [28, 29, 30].

Overall, the favorable safety profile supports advancing JBD0131 to Phase II trials in TB patients. Food‐driven PK enhancement offers a practical strategy to boost efficacy while minimizing dose‐related toxicity. If proven effective in infected populations, JBD0131 could expand therapeutic options for drug‐resistant TB—a priority per WHO guidelines.

## 4 Conclusion

JBD0131 exhibited acceptable safety and predictable PK profiles in healthy Chinese adults. The food‐enhanced bioavailability and linear accumulation suggests that further evaluation of treatment regimens is warranted.

## 5 Methods and Materials

### 5.1 Study Design

This study is a comprehensive, multicenter trial designed to evaluate the pharmacokinetics, safety, and tolerability of JBD0131 in healthy Chinese adults. The study was divided into three parts: a single ascending dose (SAD) study, a food effect study, and a multiple ascending dose (MAD) study. The Part 1 SAD study was designed to assess the safety, tolerability, and pharmacokinetics of single oral doses of JBD0131 under fasting conditions. The Part 2 food effect study evaluated the impact of food intake on the pharmacokinetics of JBD0131 using a randomized, open‐label, single‐dose, two‐period, crossover design. The Part 3 MAD study assessed the safety, tolerability, and pharmacokinetics of multiple oral doses of JBD0131 under fed conditions.

In the SAD study, six dose cohorts (20, 50, 100, 200, 300, and 400 mg) were included. Participants received a single dose of JBD0131 or placebo under fasting conditions on Day 1. PK sample collection was conducted on Days 5, 6 and 8. On Day 14, participants were contacted by phone to further assess safety and tolerability.

For food effect study, a dose of 100 mg was selected for the food effect study. Participants received JBD0131 under fasting or fed conditions on Day 1 according to randomization. PK sample collection and safety assessments were completed on Days 5, 6 and 8. After a 14‐day washout period, participants received JBD0131 under the opposite condition (fed or fasting) on Day 15 and their PK samples were collected on Days 19, 20, and 22. Participants were contacted by phone to further assess safety and tolerability on Day 28.

For MAD study, three dose groups were planned: Group 1 (100 mg BID), Group 2 (selected from 100 mg QD, 200 mg QD, or 200 mg BID based on Group 1 results), and Group 3 (selected from the same three dosing regimens based on the results of Groups 1 and 2). Participants underwent a 14‐day multiple dosing regimen from Day 1 to Day 14. In the BID group, dosing occurred twice daily from D1 to D13, with a single dose on the morning of D14. In the QD group, dosing occurred once daily from D1 to D14. PK sample collection and safety assessments were completed on Days 18, 19, and 21. Participants were contacted by phone to further assess safety and tolerability on Day 27.

### 5.2 Participants

Participants were recruited from multiple centers in China. Detailed inclusion and exclusion criteria are provided in the Supplementary Material. In summary, healthy adult male and female participants aged 18 to 45 years with a body mass index (BMI) of 18 to 24 kg/m^2^ were eligible. Male participants had to weigh at least 50 kg, and female participants at least 45 kg. During the screening period, participants underwent physical examination, vital signs monitoring, laboratory tests, 12‐lead electrocardiogram (ECG), chest X‐ray, and abdominal ultrasound (liver, gallbladder, pancreas, spleen, and kidneys). The QT corrected for heart rate by Fridericia's cube root formula (QTcF) had to be ≤470 ms, and ≤450 ms (corrected by Fridericia's formula) in female and male participants, respectively. Participants had to fully understand the purpose, nature, methods, and potential adverse reactions, voluntarily participate, and sign the informed consent form. They also had to agree to complete the study according to the protocol. The study was approved by the ethics committee following Clinical Trails Regulation of China (registration number CTR20202308 in chinadrugtrials.org.cn).

### 5.3 Treatments

In this study, a total of 104 participants were planned to be enrolled, including 8 for the blank sample collection and 96 for the formal trial. In the SAD study, all participants orally took the prescribed dose of the study drug under fasting conditions. For the food effect study, participants orally took JBD0131 tablets under fasting or after a high‐fat, high‐calorie meal as specified. In the multiple‐dose study, all participants orally took the prescribed dose of the study drug after a standard meal as specified. Each participant took the medication with approximately 240 mL of warm water.

### 5.4 Calculation of Pharmacokinetic Parameters

The relationship between dose and primary pharmacokinetic (PK) parameters (AUC and *C*_max_) was analyzed using a Power model. Metabolite identification was conducted on collected blood samples. A mixed‐effects model was employed for statistical analysis of *C*_max_, AUC_0–_ *_t_*, and AUC_0–∞_ after natural logarithmic transformation under fed and fasting conditions. In the model, treatment group (fasting and fed), treatment sequence, and treatment period were fixed effects, while participants nested within sequences were random effects. The model‐estimated adjusted mean differences (fed—fasting) and their 90% confidence intervals were back‐transformed to obtain the geometric mean ratios (fed/fasting) and their 90% confidence intervals for the respective PK parameters. If the 90% confidence intervals of the geometric mean ratios for *C*_max_, AUC_0–_ *_t_*, and AUC0_0–∞_ entirely fell within the range of 80.00% to 125.00%, it was concluded that there was no significant difference in the primary pharmacokinetic parameters between the two dosing conditions, indicating that food had no significant impact on the pharmacokinetics of JBD0131.

### 5.5 Sample Size Determination

A total of 104 subjects were planned for enrollment in this study, comprising 8 subjects for the blank sample collection trial and 96 subjects for the treatment group. During the Phase I clinical study, subject enrollment in respective dose groups could be appropriately supplemented to account for potential dropouts. The study ultimately included only two dose groups due to adjustments in the dosing regimen during the multiple‐dose phase. Furthermore, one subject in the multiple‐dose phase was replaced due to withdrawal prior to the first dose administration. Consequently, a total of 95 subjects were actually enrolled, which included 8 subjects for the blank sample collection trial and 87 subjects for the treatment group.

### 5.6 Safety Statistical Analysis

Based on the safety analysis set, the dosing information, incidence of adverse events, physical examination findings, vital signs, 12‐lead electrocardiogram results, laboratory tests, blood potassium measurements, ophthalmological examinations, and depression scale scores, along with their changes from baseline, were summarized and subjected to descriptive statistics for subjects in this trial. Adverse events were coded and classified using the Medical Dictionary for Regulatory Activities (MedDRA), version 25.0, according to the System Organ Class (SOC) and Preferred Term (PT).

## Author Contributions

Xiawei Wei conceived and supervised the study and designed the experiments with initial drafting of the manuscript. Jia Miao and Zhenlin Wang contributed were responsible for the study design, data collection, and initial drafting of the manuscript. Zhenyu Ding, Huashan Shi, Yongping Qin, and Tiantao Gao were involved in the execution of the study, including patient recruitment and data analysis. Ning Jiang provided critical revisions to the manuscript for important intellectual content and obtained funding. Jianqing He and Manni Wang contributed to the conception of the study. All authors have read and approved the final manuscript.

## Ethics Statement

This study is approved by Clinical Trial Ethics Review Committee of West China Hospital, Sichuan University (CXHL1700246, CXHL1700247).

## Funding

This work was supported by the Sichuan Province Distinguished Young Science and Technology Program (2019JD100008, X.W.).

## Conflicts of Interest

Jumbo Drug Bank Co. Ltd. provided the JBD0131 and financial support to this clinical trial. Ning Jiang and Xiawei Wei are employees of this Company and declare a potential conflict of interest. The other authors declare that they have no competing interests.

## Supporting information



Supporting Information

## Data Availability

Upon reasonable request from corresponding authors, the data will be available upon reasonable request from corresponding authors after 5 years from the publication.
