# Supplementary material for: Safety, Tolerability, and Pharmacokinetics of a Novel Nitroimidazooxazole Antitubercular Agent in Healthy Adults: A Phase I Trial
Source: MedComm (2020). 2025 Dec 4;6(12):e70510. doi: 10.1002/mco2.70510 (PMC12678841; doi:10.1002/mco2.70510)
Supplement: Supplementary file 1 — Supporting Information [file MCO2-6-e70510-s001.docx]

**Safety, tolerability, and pharmacokinetics of JBD0131 tablets，a novel nitroimidazooxazole antitubercular agent， following single and multiple ascending doses with food effect assessment in healthy adults: a phase I trial**

Jia Miao^2^*^#^, Zhenling Wang^1#^, Zhenyu Ding^3^, Huashan Shi^3^, Yongping Qin^2^, Tiantao Gao^2^, Ning Jiang^5^ , Jianqing He^4^*, Manni Wang^3^*, Xiawei Wei^1,5^*

^1^ Laboratory of Aging Research and Cancer Drug Target, National Clinical Research Center for Geriatrics, State Key Laboratory of Biotherapy and Cancer Center, West China Hospital, Sichuan University, Chengdu, China.

^2^ GCP Center/Institute of Drug Clinical Trials, West China Hospital, Sichuan University, Chengdu, Sichuan, China.

^3^ Department of Biotherapy, Cancer Center and State Key Laboratory of Biotherapy, West China Hospital, Sichuan University, Chengdu 610041, Sichuan, China

^4^ Department of Pulmonary and Critical Care Medicine, State Key Laboratory of Respiratory Health and Multimorbidity, West China Hospital, Sichuan University, Chengdu, 610041, China.

^5^ Jumbo Drug Bank Co., Ltd., Chengdu, China

**^#^** These authors contributed equally to this work.

***Correspondence**

Jia Miao; Email: miaosiyi1971@163.com

Jianqing He; Email: jianqing_he@scu.edu.cn

Manni Wang; Email: wangmanni@scu.edu.cn

Xiawei Wei; Email: [xiaweiwei@scu.edu.cn](mailto:xiaweiwei@scu.edu.cn)

**Supplementary Table 1. Summary of adverse events in the single ascending dose (SAD) study.**

|  | **20mg**  **(N=3)** | **50mg**  **(N=8)** | **100mg**  **(N=8)** | **200mg**  **(N=8)** | **300mg**  **(N=8)** | **400mg**  **(N=8)** | **Placebo**  **(N=11)** |
| --- | --- | --- | --- | --- | --- | --- | --- |
| **Incidence, Number of Events , (%)** | | | | | | | |
| AEs | 0 0 | 12 5 (62.5) | 12 6 (75.0) | 8 5 (62.5) | 10 5 (62.5) | 3 3 (37.5) | 9 6 (54.5) |
| Drug-related Adverse Reactions | 0 0 | 6 5 (62.5) | 7 6 (75.0) | 2 2 (25.0) | 8 5 (62.5) | 1 1 (12.5) | 8 6 (54.5) |
| SAEs | 0 0 | 0 0 | 0 0 | 0 0 | 0 0 | 0 0 | 0 0 |
| SAEs among Drug-related Adverse Reactions | 0 0 | 0 0 | 0 0 | 0 0 | 0 0 | 0 0 | 0 0 |
| AEs Leading to Early Termination of Trial | 0 0 | 0 0 | 0 0 | 0 0 | 0 0 | 0 0 | 0 0 |

Abbreviation: adverse events (AEs); serious adverse events (SAEs).

**Supplementary Table 2. Summary of adverse events in the food-effect study.**

|  | **Fasting**  **(N=12)** | **Postprandial**  **(N=12)** | **Total**  **(N=12)** |
| --- | --- | --- | --- |
|  | **Incidence, Number of Events , (%)** | | |
| AEs | 11 8 (66.7) | 14 9 (75.0) | 25 12 (100) |
| Drug-related Adverse Reactions | 10 8 (66.7) | 10 7 (58.3) | 20 11 (91.7) |
| SAEs | 0 0 | 0 0 | 0 0 |
| SAEs among Drug-related Adverse Reactions | 0 0 | 0 0 | 0 0 |
| AEs Leading to Early Termination of Trial | 0 0 | 0 0 | 0 0 |

Abbreviation: adverse events (AEs); serious adverse events (SAEs).

**Supplementary Table 3. Summary of clinically significant laboratory abnormalities in food-effect studies.**

|  | **Fasting to Postprandial**  **(N=6)** | | **Postprandial to Fasting**  **(N=6)** | | **Total**  **(N=12)** | |  |
| --- | --- | --- | --- | --- | --- | --- | --- |
| **Laboratory Test（Unit）** | **Incidence, Number of Events (%)** | | | | | |  |
| **Blood Routine** | 0 | 0 | 10 | 1 (16.7) | 10 | 1 (8.3) | |
| Hemoglobin (g/L) | 0 | 0 | 10 | 1 (16.7) | 10 | 1 (8.3) | |
| **Blood Biochemistry** | 25 | 3 (50.0) | 6 | 3 (50.0) | 31 | 6 (50.0) | |
| Amylase (U/L) | 17 | 2 (33.3) | 1 | 1 (16.7) | 18 | 3 (25.0) | |
| Alanine Aminotransferase (U/L) | 6 | 1 (16.7) | 0 | 0 | 6 | 1 (8.3) | |
| Creatinine (μmol/L) | 0 | 0 | 4 | 1 (16.7) | 4 | 1 (8.3) | |
| Potassium (mmol/L) | 2 | 2 (33.3) | 1 | 1 (16.7) | 3 | 3 (25.0) | |
| **Blood cortisol** | 2 | 1 (16.7) | 2 | 1 (16.7) | 4 | 2 (16.7) | |
| Cortisol (nmol/L) | 2 | 1 (16.7) | 2 | 1 (16.7) | 4 | 2 (16.7) | |
| **Stool Routine** | 9 | 3 (50.0) | 8 | 5 (83.3) | 17 | 8 (66.7) | |
| Occult Blood | 7 | 3 (50.0) | 8 | 5 (83.3) | 15 | 8 (66.7) | |
| Red Blood Cells / (HPF) | 2 | 2 (33.3) | 0 | 0 | 2 | 2 (16.7) | |

**Supplementary Table 4. Summary of adverse events in the multiple ascending dose (MAD) study.**

|  | **100mg BID**  **(N=8)** | **200mg BID**  **(N=8)** | **Placebo**  **(N=4)** | **Total**  **(N=20)** |
| --- | --- | --- | --- | --- |
|  | **Incidence, Number of Events , (%)** | | | |
| AEs | 25 8 (100) | 32 8 (100) | 8 4 (100) | 65 20 (100) |
| Drug-related adverse reactions | 17 7 (87.5) | 22 7 (87.5) | 5 3 (75.0) | 44 17 (85.0) |
| SAEs | 0 0 | 0 0 | 0 0 | 0 0 |
| SAEs among Drug-related Adverse Reactions | 0 0 | 0 0 | 0 0 | 0 0 |
| AEs Leading to Early Termination of Trial | 0 0 | 0 0 | 0 0 | 0 0 |

Abbreviation: adverse events (AEs); serious adverse events (SAEs).

**Supplementary Table 5. Summary of pharmacokinetic parameters of WXWH0131 in multiple-dose group D1.**

| PK  parameters |  | 100 mg BID  (N=8) | 200 mg BID  (N=8) |
| --- | --- | --- | --- |
| C_max_(ng/mL) | Mean±SD | 302.8505±80.4758 | 458.9593±81.7184 |
| T_max_(h) | Median (Min, Max) | 3.25（2.50, 5.00） | 3.25（1.50, 4.00） |
| AUC_0-12_(h*ng/mL) | Mean±SD | 1811.3126±460.6653 | 3251.4226±613.7995 |

Abbreviations: Pharmacokinetics (PK), maximum concentration (Cmax), time to maximum concentration (Tmax), area under the concentration-time curve (AUC), area under the curve from 0 to 12 hours (AUC0-12).

**Supplementary Table 6. Summary of pharmacokinetic parameters of WXWH0131 in multiple-dose group D14.**

| PK  parameters |  | 100 mg BID  (N=8) | 200 mg BID  (N=8) |
| --- | --- | --- | --- |
| T_max,ss_(h) | Median (Min, Max) | 3.75（2.50, 5.00） | 3.75（2.50, 4.00） |
| C_max,ss_(ng/mL) | Mean±SD | 453.2094±83.6371 | 777.6784±117.7175 |
| C_min,ss_(ng/mL) | Mean±SD | 224.3113±56.1328 | 358.5413±81.8357 |
| C_avg,ss_(ng/mL) | Mean±SD | 316.3235±62.8671 | 522.1710±92.0925 |
| AUC_0-12,ss_(h*ng/mL) | Mean±SD | 3795.8823±754.4047 | 6266.0524±1105.1100 |
| AUC_0-t,ss_(h*ng/m L) | Mean±SD | 8159.3545±2468.3919 | 13230.6377±3641.4755 |
| AUC_0-∞,ss_(h*ng/mL) | Mean±SD | 8206.1242±2484.2879 | 13270.1560±3640.5313 |
| t_1/2,ss_ (h) | Mean±SD | 11.84±3.56 | 11.41±2.12 |
| V_d_/F_,ss_ (L) | Mean±SD | 454.3922±122.2207 | 539.1159±148.7892 |
| CL/F_,ss_ (L/h) | Mean±SD | 27.4217±6.3349 | 32.8077±5.8231 |
| Rac(C_max_) | Mean±SD | 1.5425±0.2402 | 1.7366±0.3827 |
| Rac(AUC) | Mean±SD | 2.1455±0.3661 | 1.9726±0.4343 |
| DF(%) | Mean±SD | 73.68±9.90 | 81.43±8.84 |

Abbreviations: Pharmacokinetics (PK), area under the concentration-time curve during a 12-hour dosing interval at steady state (AUC₀₋₁₂,ss), area under the concentration-time curve from time zero to the last measurable concentration at steady state (AUC₀₋t,ss), area under the concentration-time curve from time zero to infinity at steady state (AUC₀₋∞,ss), Bis In Die (BID), apparent clearance divided by bioavailability at steady state (CL/F,ss), maximum concentration at steady state (Cmax,ss), minimum concentration at steady state (Cmin,ss), average concentration over a dosing interval at steady state (Cavg,ss), degree of fluctuation (DF), accumulation ratio based on Cmax (Rac(Cmax)), accumulation ratio based on AUC (Rac(AUC)), terminal elimination half-life at steady state (t₁⁄₂,ss), apparent volume of distribution divided by bioavailability at steady state (Vd/F,ss).

**Supplementary Table 7. Summary of pharmacokinetic parameters of WXWD in multiple-dose group D1.**

| PK  parameters |  | 100 mg BID  (N=8) | 200 mg BID  (N=8) |
| --- | --- | --- | --- |
| C_max_(ng/mL) | Mean±SD | 20.8905±4.4817 | 48.4458±13.0187 |
| T_max_(h) | Median (Min, Max) | 4.00（3.00, 5.00） | 3.75（3.50, 4.00） |
| AUC_0-12_(h*ng/mL) | Mean±SD | 132.9229±32.7152 | 322.4667±89.2238 |

Abbreviations: Pharmacokinetics (PK), maximum concentration (Cmax), time to maximum concentration (Tmax), area under the concentration-time curve (AUC), area under the curve from 0 to 12 hours (AUC0-12).

**Supplementary Table 8. Summary of pharmacokinetic parameters of WXWD in multiple-dose group D14.**

| PK  parameters |  | 100 mg BID  (N=8) | 200 mg BID  (N=8) |
| --- | --- | --- | --- |
| T_max,ss_(h) | Median (Min, Max) | 4.00（2.50, 6.00） | 4.01（3.50, 12.00） |
| C_max,ss_(ng/mL) | Mean±SD | 131.7666±23.9929 | 238.6320±43.0477 |
| C_min,ss_(ng/mL) | Mean±SD | 104.7376±22.8390 | 178.7654±33.8929 |
| C_avg,ss_(ng/mL) | Mean±SD | 115.9940±21.7211 | 204.8761±38.9693 |
| AUC_0-12,ss_(h*ng/mL) | Mean±SD | 1391.9278±260.6528 | 2458.5128±467.6311 |
| AUC_0-t,ss_(h*ng/m L) | Mean±SD | 7457.2772±2458.6804 | 12035.6795±2902.6639 |
| AUC_0-∞,ss_(h*ng/mL) | Mean±SD | 7932.1383±2758.1643 | 12522.3144±3113.6237 |
| t_1/2,ss_ (h) | Mean±SD | 37.89±8.13 | 33.76±6.99 |
| DF(%) | Mean±SD | 23.95±5.89 | 29.47±4.00 |

Abbreviations: Pharmacokinetics (PK), area under the concentration-time curve during a 12-hour dosing interval at steady state (AUC₀₋₁₂,ss), area under the concentration-time curve from time zero to the last measurable concentration at steady state (AUC₀₋t,ss), area under the concentration-time curve from time zero to infinity at steady state (AUC₀₋∞,ss), Bis In Die (BID), apparent clearance divided by bioavailability at steady state (CL/F,ss), maximum concentration at steady state (Cmax,ss), minimum concentration at steady state (Cmin,ss), average concentration over a dosing interval at steady state (Cavg,ss), degree of fluctuation (DF), accumulation ratio based on Cmax (Rac(Cmax)), accumulation ratio based on AUC (Rac(AUC)), terminal elimination half-life at steady state (t₁⁄₂,ss), apparent volume of distribution divided by bioavailability at steady state (Vd/F,ss).
